# Review: Understanding Rare Genetic Diseases in Low Resource Regions Like Jammu and Kashmir – India

**DOI:** 10.3389/fgene.2020.00415

**Published:** 2020-04-30

**Authors:** Arshia Angural, Akshi Spolia, Ankit Mahajan, Vijeshwar Verma, Ankush Sharma, Parvinder Kumar, Manoj Kumar Dhar, Kamal Kishore Pandita, Ekta Rai, Swarkar Sharma

**Affiliations:** ^1^Human Genetics Research Group, School of Biotechnology, Shri Mata Vaishno Devi University, Katra, India; ^2^Bioinformatics Infrastructure Facility, School of Biotechnology, Shri Mata Vaishno Devi University, Katra, India; ^3^Shri Mata Vaishno Devi Narayana Superspeciality Hospital, Katra, India; ^4^Institute of Human Genetics, University of Jammu, Jammu, India; ^5^Independent Researcher, Health Clinic, Jammu, India

**Keywords:** rare diseases, Next-Generation DNA Sequencing, endogamy, consanguinity, India, Jammu and Kashmir, Bottom-up Approach

## Abstract

Rare diseases (RDs) are the clinical conditions affecting a few percentage of individuals in a general population compared to other diseases. Limited clinical information and a lack of reliable epidemiological data make their timely diagnosis and therapeutic management difficult. Emerging Next-Generation DNA Sequencing technologies have enhanced our horizons on patho-physiological understanding of many of the RDs and ushered us into an era of diagnostic and therapeutic research related to this ignored health challenge. Unfortunately, relevant research is meager in developing countries which lack a reliable estimate of the exact burden of most of the RDs. India is to be considered as the “Pandora’s Box of genetic disorders.” Owing to its huge population heterogeneity and high inbreeding or endogamy rates, a higher burden of rare recessive genetic diseases is expected and supported by the literature findings that endogamy is highly detrimental to health as it enhances the degree of homozygosity of recessive alleles in the general population. The population of a low resource region Jammu and Kashmir (J&K) – India, is highly inbred. Some of its population groups variably practice consanguinity. In context with the region’s typical geographical topography, highly inbred population structure and unique but heterogeneous gene pool, a huge burden of known and uncharacterized genetic disorders is expected. Unfortunately, many suspected cases of genetic disorders remain undiagnosed or misdiagnosed due to lack of appropriate clinical as well as diagnostic resources in the region, causing patients to face a huge psycho-socio-economic crisis and many a time suffer life-long with their ailment. In this review, the major challenges associated with RDs are highlighted in general and an account on the methods that can be adopted for conducting fruitful molecular genetic studies in genetically vulnerable and low resource regions is also provided, with an example of a region like J&K – India.

## Introduction

Rare diseases (RDs) are progressive, chronically debilitating and/or life-threatening heterogeneous clinical conditions that affect a limited fraction of individuals from the general population in comparison to other prevalent diseases (Schieppati et al., 2008; Richter et al., 2015; Danese and Lippi, 2018). However, these have been recently recognized as one of the major public health concerns prevalent across the globe. It has been estimated that RDs are altogether afflicting a significantly larger proportion of the global population (translating to billion individuals) (Schieppati et al., 2008). There are several challenges that make diagnosis and therapeutic management of RDs cumbersome and impede RDs-related research. Despite several global initiatives to address the RDs-associated challenges, a lot of work needs to be carried out in order to deal with this ignored health sector. In a developing country like India, the RD-related research has been hampered by limited advanced clinical resources and far-to-approach, sporadically localized genetic services centers (Pradhan et al., 2011; Aggarwal and Phadke, 2015; Kasthuri, 2018).

India’s primitive migration history and highly diverse population architecture which is epitomized by socio-cultural, geographical, linguistic, and religious isolation have been suggested as the main contributor to the country’s genetic diversity (Basu et al., 2016). However, biological isolation of several endogamous population groups might have resulted in a relatively higher prevalence of genetic disorders in India (Pradhan et al., 2011). One of India’s highly diverse and conglomeration of various inbred population groups, the population of Jammu and Kashmir (J&K) region, is expected to be an unexplored reservoir of genetic disorders. The J&K population is characterized by several endogamous groups with specific marital affinities, including the practice of consanguinity. In addition, the geographical isolation of various groups due to complex terrain may provide J&K a unique genetic architecture and disease profile. Unfortunately, the region has largely remained unexplored in context of genetic-based research. This has potentially contributed to the ignorance of a large number of hereditary diseases which are likely restricted to specific extended families or communities on a whole. Advanced high-throughput genomics-based approaches have, however, outpaced genetic research in J&K and precise diagnosis of some monogenic disorders, which are otherwise rare in prevalence across the globe but are likely to have attained higher incidence in the region due to higher inbreeding rates (Rai et al., 2016; Kuchay et al., 2019). We propose that the population of J&K, thus, offers a special niche to understand yet-to-be-explored underpinning molecular etiology of genetic diseases. With respect to the population of J&K, genetic research holds a huge potential in designing diagnostic protocols for prevailing genetic diseases and development of therapeutics.

This review provides a brief account on RDs and their prevalence, followed by a discussion on the major RDs-associated challenges in general, an account on the methods that can be adopted for conducting fruitful molecular genetic studies of monogenic diseases, and the experiences of genetic research in Indian context with a special reference to a genetically vulnerable and low resource region like J&K.

## Rare Diseases: Some Facts

Rare diseases are caused by function-altering variation(s) in a single gene, and hence are referred to as “single gene disorders” or “monogenic disorders” (Boycott et al., 2017). There is no single universal definition for prevalence of known RDs. The base prevalence rate of RDs set by the World Health Organization (WHO) is approximately 1 in 2,000 people (Lopes et al., 2018). However, different nations have their own definitions for the prevalence of RDs which is mostly based on the prevalence of a disease in their own population, status of health care system and availability of resources. A genetic disorder prevalent in the European Union (EU) is considered rare only if it affects 5 or less per 10,000 cases, whereas the incidence rate for RDs in the United States is 7 or less per 10,000 individuals (Lang and Wood, 1999; Hughes et al., 2005). These numbers translate to nearly 30 million Europeans and 25 million North Americans (approximately 1 in every 10) affected by any of the known RDs (Haffner et al., 2002; Wastfelt et al., 2006). The incidence rate is estimated to be ≤2.5 cases in 10,000 -and 1 in 10,000 individuals for Japan and Australia, respectively (Lang and Wood, 1999; Hughes et al., 2005; Zurynski et al., 2008).

Nearly 7,000 distinct RDs have been delineated and new ones are being consistently reported in the literature (Ng et al., 2010b). It is believed that a majority of RDs (80% or more) are genetic in origin, whereas distinct underpinning causes for the remaining disorders are not well understood (Mckusick, 2007; Song et al., 2012a). The remaining RDs may be caused by environmental (for instance, Jamaican vomiting sickness, mesothelioma), infectious (for instance, maternofetal measles) or immunological (for instance, juvenile chronic arthritis) factors (Guillem et al., 2008; Lopes et al., 2018). There is a wide variability in severity and expression of distinct RDs. Many of these are congenital on onset and continue to exist with poor prognosis (lifelong-disability and/or early death) over the lifetime of the afflicted individual, while in some individuals the symptoms of the disease (whether same or other) may appear later in life, thus, presenting difficulties in their diagnosis. It has been estimated that nearly 50% of reported RDs occur in children, 30% of RDs patients die during infancy (before the age of 5), and 12% of them die between 5 and 15 years of age (Song et al., 2012b).

Although RDs are distinctly defined as rare on the basis of their low prevalence, yet their cumulative burden on the public health is huge. The known RDs altogether affect a substantial number of estimated 350 million individuals across the globe which translates to approximately 10% of the global population (Song et al., 2012b; Boycott et al., 2013). About 80% of them are cumulatively affected by merely lesser than 100 known RDs (Luzzatto et al., 2015). Nonetheless, the available figures for their global prevalence are alarming indicating that these altogether affect more individuals than those suffering with common diseases; for instance diabetes has an estimated incidence of 20.8 million and 1.4 million among Americans and Australians, respectively (Dunstan et al., 2002; Zurynski et al., 2008).

## Challenges Associated With RDs and Related Progress

Different types of RDs altogether constitute a major exigent global public-health issue and present several formidable challenges which are largely related to diagnosis, access to health care services and disease-specific interventions, lack of specialized clinical personnel and specific infrastructure, challenges faced by the RD patients and their families, and lack of ample resources for RD-associated research and development (R&D). Further these challenges inevitably result in unreliability of the available patient-registries and, therefore, vague epidemiological data. To address these challenging issues, a drive toward universal health coverage is required so that the needs of RD patients get fulfilled and public and government (national or international) agencies invest their funds into fundamental biomedical research for understanding the etiology of diseases and discovering their novel therapeutic targets and strategies. However, a significant scientific and technological progress has been witnessed over the last few decades that have notably filled our knowledge gaps on the understanding of several RDs and made their diagnosis and management a bit simple task. A brief account on major RDs-associated challenges and their related progress has been provided in this section.

### Clinical Challenges, Lack of Reliable Patient-Registries and Inaccurate Epidemiological Data

Clinical challenges include limited or no availability of appropriate clinical resources, lack of specific literature and evidence-based knowledge or difficulty in assessing knowledge sources, and has rendered RDs diagnosis and management troublesome (Schieppati et al., 2008). Inadequate clinical resources include lack of clinicians/experts having a sound knowledge and experience in Clinical Genetics, lack of standardized clinical guidelines and specific clinical infrastructure which, thereby, usually require the needy patients to undergo unavoidable clinical investigations that end up getting them multiple indefinite diagnoses (Knight and Senior, 2006; Zurynski et al., 2008). It is obvious that a clinician’s disease management expertise is proportional to the frequency of clinician-patient encounters. Since RDs have a relatively less frequency, such clinical encounters are assumed to be negligible which subsequently contributes to a huge clinical knowledge gap. This knowledge gap is also contributed by insufficient availability of sources of clinical information on the underpinning cause, patho-physiology and natural course of most of the RDs, and limited availability and endorsement of relevant guidelines of the allied clinical societies. Clinical heterogeneity of distinct RDs results in many subtypes leading to different clinical manifestations and course which along with lack of relevant clinical information usually make the diagnosis of several RDs troublesome. No diagnosis or misdiagnosis to the patients ultimately result in no or lack of reliable patient-registries which subsequently ends up in the lack of an accurate epidemiological data on RDs (Zurynski et al., 2008). Unfortunately, of the estimated ≥7,000 disorders defined as “single gene disorders,” a detailed phenotypic information on about 5,551 of these have been currently reported in the Online Mendelian Inheritance in Man^®^ database (OMIM ^®^),^1^ while phenotypic information on the remaining disorders is scanty till now. According to the data available from the Orphanet database,^2^ epidemiology of only 29% of ≥7,000 RDs has been reported.

### Problems Faced by the Patients and Their Families

RDs patients and their families face huge psycho-socio-economic burden due to social isolation, difficulty in accessing appropriate health care services, delay in diagnosis, and uncertainty about their future and financial hardships. Most of the RDs are often severely disabling, impair the overall abilities of the patients, and substantially reduce the quality of their life and life expectancy. About half of the RDs appear in early childhood which makes it hard or impossible for the young patients to attain their education in the schools or colleges (Zurynski et al., 2008). The patients and their families also have to experience social stigma in the form of social isolation and overall discrimination. Due to fear of social stigma compounded by lack of awareness on their health condition (whether being an inherited or genetic disorder), many a times patients intentionally do not get a clinical consultation. This, in turn, directly impacts the reliability of the patient-registries since these patients do not get registered in the hospitals. Patients generally struggle to find specialized clinicians having sound knowledge and experience in Clinical Genetics. Clinicians having deep knowledge and expertise in management of RDs are usually concentrated in geographically dispersed specialized centers which may remain beyond the patient’s access or require most of the patients to travel long distances or to shift their residence to a new place for getting a diagnosis (Zurynski et al., 2008). These factors altogether result in diagnostic delays, misdiagnosis or no diagnosis and ultimately no effective treatments to the patients. All this complicates the medical condition of the patients as they are only left with an option of suffering with the primary or secondary consequences of their disease and its late sequelae (Yang et al., 2013). Besides, a long-term search for an accurate diagnosis of RDs, referred to as the “diagnostic odyssey,” usually incur a huge medical expenditure with unsuccessful attempts and consumption of limited resources which has its own financial implications on the patient’s family as raising a disabled child is relatively expensive than for a normal child (Zurynski et al., 2008; Yang et al., 2013).

### Diagnostic Challenges of Rare Diseases

Establishing the precise diagnoses for RDs is usually difficult. Their diagnosis is highly dependent on the access to diagnostic testing and requires determination of the underpinning genetic cause (Boycott and Ardigo, 2018). Factors including clinical heterogeneity, co-morbidity and varying disease course among different RDs patients highly demand a differential diagnosis of the disease with which they suffer life-long (Romdhane et al., 2016; Benjamin et al., 2017). However, establishing differential diagnosis is a meticulous and time-consuming task incurring a diagnostic odyssey that usually relies on the skills of concerned clinician and the diagnostic tests that a patient has to undergo, as discussed earlier.

Since 2010, Next-Generation Sequencing (NGS) has accelerated the rate of RDs diagnosis. Although NGS has significantly accelerated the rate of precision diagnoses in RDs patients, but with a diagnostic yield of only 25–50% (Li et al., 2018). For the remaining significant fraction of patients comprising of the ones presenting complex phenotypes, it fails to yield any confirmed diagnosis due to several technical limitations (Wenger et al., 2017). However, many approaches pertaining to genetic diagnosis have recently emerged. An amalgamation of comparative reanalysis of clinical as well as non-clinical NGS data using various newly emerged data analysis pipelines and software in consideration with updated scientific literature can be employed for improving the diagnostic yield through NGS. For instance, a recent study on reanalysis of 40 unsolved exome reports later led to a precise diagnosis in about 10% of cases (Wenger et al., 2017).

### Challenges Faced in RD-Related R&D and Therapeutics

The RDs-related R&D is highly complicated and impeded by several challenging issues including a huge knowledge gap about the underpinning causes of distinct RDs, lack of an international standard code for their classification, assembling cohorts of patients for conducting a research study owing to their distinct rare prevalence, and insufficient funding opportunities on RDs-research. These challenges, further, compound the determination of suitable therapeutic interventions and development of particular drug molecules for targeting a specific clinical condition. No single institution and/or country have a sufficient figure on the number of affected individuals for carrying out a generalized clinical and translational research. This could be mainly attributed to the International Classification of Diseases (ICD) system used in many countries for disease classification. The ICD is not suitable for most of the RDs which further hampers inclusion of national and international patients’ registries into reliable epidemiological databases and lead to non-reliable assessment of their economic and social burden (Schieppati et al., 2008). The other major reason is that some RDs occur so infrequently (<1 in 1,000,000 population) that only by conducting international population-based study can sufficient numbers of geographically dispersed patients be accrued for a clinical investigation so that a higher power study could be yielded. Recruitment of such a number of patients into a research study is further impeded by the lack of reliable patient registries which subsequently lead to non-reliable assessment of disease burden, imprecise cost estimations of resource consumption involved in the whole process of research, drug development and clinical trials for developing a suitable disease management or therapeutic strategy, and missing out a potential funding opportunity (Schieppati et al., 2008). Funding and policy-making has also been a major obstacle in establishing infrastructure for maintaining registries of the patients (Forrest et al., 2011). Although for some of the disorders, national and/or international patient registries have been regularly maintained by different associations, yet there is no recognition for most of these at the Government level due to lack of or limited documentation of RDs patients in the local hospitals (Schieppati et al., 2008).

Taking into consideration of a dire need for the formulation of a universal RDs classification system that would provide comprehensive information on known RDs, the European Rare Disease Task Force of the Health and Consumers Protection Directorate General of the European Commission in collaboration with WHO has set up the ICD-10. Besides, several other classification systems like the Orphanet, the OMIM ^®^, the Systemized Nomenclature of Medicine – Clinical Terms (SNOMED-CT) are available for coding of these diseases, with each of these have their own advantages and disadvantages (Ayme et al., 1998; Mckusick, 2007; De Silva et al., 2011). Another international group called the “Rare Disease Terminology and Definitions Used in Outcomes Research Working Group” under aegis of the “International Society for Pharmacogenomics and Outcomes Research (ISPOR) Rare Disease Special Interest Group” has been established for the development of a universal RDs definition (Richter et al., 2015). With regular up-gradation of clinical infrastructure and updating of clinical databases via collaborative efforts, knowledge of these diseases is also improving. Such an increase in focus over RDs has been mainly facilitated by the relentless work of a significant number of legislations, NGO committees and patients’ organizations which have highlighted the plight of RDs patients and stressed over their timely therapeutic management. This has further paved the impetus to RDs research by incentivizing pharmaceutical and biotechnology companies, and in turn, has proven instrumental in figuring out diagnostic, effective therapeutics and preventative modalities for a variety of RDs (Richter et al., 2015). Unless before the US Orphan Drug Act (1983) and the European Union (EU) Regulation 142/2000 (2000) on medicinal products came into effect, the pharmaceutical industry was ignorant to the development of “orphan drugs” or drugs for the treatment of distinct RDs (Haffner et al., 2002; Richter et al., 2015; Auvin et al., 2018). With their enactment, incentives were provided to pharmaceutical companies for the development of RDs diagnostics and therapeutic strategies in the United States and the EU, and since then, their success could be defined by the United States Food and Drug Administration (FDA) and the European Medicine Agency (EMA) approval for marketing of several hundreds of therapeutic drugs and biological products for the treatment of merely 5% RDs mainly including rare forms of cancers (Haffner et al., 2002; Haffner, 2006; Braun et al., 2010; Richter et al., 2015; Auvin et al., 2018). Although these approved drugs have significantly transformed the treatment of only 5% of these diseases for which fewer or no treatment options were available earlier, yet there is a lack of availability of treatment options for a significantly higher percentage (95%) of RDs. Since, the available drugs are extremely expensive, they pose a huge financial stress on the affected families, health-care systems and donor agencies.

However, for creating an advanced integrated research pipeline for the development of novel therapeutics for RDs, several governmental and non-governmental organizations and their programs including the United States National Institutes of Health’s (NIH) “Therapeutics for Rare and Neglected Diseases (TRND)” program, the “Genetic and Rare Diseases (GARD) Information Center” (a collaborative effort of two NIH centers namely the National Human Genome Research Institute (NHGRI) and the National Center for Advancing Translational Sciences (NCATS)), the NIH’s “Office of Rare Diseases Research” (ORDR), the “National Organization for Rare Diseases” (NARD), the “European Organization for Rare Diseases” (EURORDIS), the “International Rare Diseases Research Consortium” (IRDiRC), the “Genetic Alliance,” the “Vereniging Spierziekten Nederland” (VSN), etc., have been initiated during the past few years for advocating RDs patients’ need for national/international RDs policies. These initiatives are providing a platform for patients’ advocacy, research funding and development of new advanced amenities for addressing the challenges pertaining to ignored RDs with a common aim of dissemination of the related data and information to the scientific community and demonstration of their overall usefulness in RDs diagnostics and therapeutics. Despite, it is assumed that the current rate of R&D would not be able to generate therapeutics for most of the RDs for the next several years. This universal RD-challenge would only be addressed by an unprecedented, large scale international cooperation between different geographically scattered government and non-government agencies and R&D units of different pharmaceutical companies.

## Understanding Rare Genetic Disorders: Methodologies for Studying Molecular Etiology

In general, the first-line diagnostics includes detection of pathognomonic phenotypic changes, disease-phenotype correlation and biochemical analysis of the known disease biomarkers through newborn disease screening methods, hematological evaluation, metabolic testing, and radiographic examinations. Once a preliminary diagnosis is established, its authenticity primarily relies on the determination of molecular etiology of query disease through genetic screening of the patients. Different genetic screening methods include traditional as well as advanced cytogenetic techniques, single-gene sequencing, and sequencing of a panel of genes associated with specific disease types. Though each of these have their own limitations, yet are widely practiced for the diagnosis of genetic diseases in many countries and are helping risked families in early detection of a possible disease, its early intervention and preventative/palliative care.

For many previous years, underpinning genetic reasons for several human diseases have been primarily deciphered through linkage and association-based genetic epidemiological studies. However, with further advancements in several biological techniques, genetic epidemiological studies have seen a dramatic shift from conventional study approaches (such as twin studies, family-based linkage studies) to population-based genome-wide association studies (GWAS) to the studies based on the most-advanced Next-Generation DNA Sequencing (NGS) technologies. On one hand where population-based study designs like GWAS and Twin-based Epidemiological Studies remain uninformative in understanding RDs, specific methodologies to facilitate determination of their distinct molecular etiology also exist but are cumbersome. With advent of new, high-throughput technologies, these efforts have improved significantly. Brief accounts of these study designs are provided below:

### Genetic Linkage Studies

Genetic linkage studies are generally used for mapping/determining the most probable chromosomal/genomic loci co-segregating with a disease phenotype through studies either based on the information of disease’s mode of inheritance (dominant or recessive) in the family pedigrees (parametric or model-based linkage analysis) or on some genome-wide polymorphic genetic markers such as microsatellites in the selected families (non-parametric or model-free linkage analysis) (Dawn Teare and Barrett, 2005; Teare and Santibanez Koref, 2014). The main principle of these family-based studies is that physically close genetic loci on a chromosome remain highly linked during meiosis (that is, the probability of recombination between them is <50%) and are inherited independently from parents to offspring (Pulst, 1999; Dawn Teare and Barrett, 2005). If a set of variations are linked together in a same haplotype in a particular population, they are considered to be in “linkage disequilibrium” (LD) and the genetic loci in LD are considered to be linked (Dawn Teare and Barrett, 2005). Studies based on linkage mapping in affected and unaffected siblings or affected individuals and their parents (child-parent trio) in multi-case extended families (loaded families) or pedigrees provide information on the co-segregation of genetic variations with the disease phenotype, and minimize genotyping error and maximize power for assertion (Whittemore and Nelson, 1999; Bailey-Wilson and Wilson, 2011; Barrett, 2014). In the first course of a genetic characterization of a clinical phenotype or trait for which exact causal genetic component is unknown, linkage studies are extremely useful in the identification of a genetic locus that might be harboring a disease-causing gene being shared between affected individuals and needs just a very few markers to carry out the same. However, this does not mean that linkage studies could map the causative gene alone. For this purpose, these mostly rely on “positional cloning” workflows (Teare and Santibanez Koref, 2014). The other limitation of linkage studies is that the power of a linkage study may get highly reduced when both incomplete penetrance and locus heterogeneity exists in the study subjects (Teare and Santibanez Koref, 2014).

Linkage studies are the most powerful tool in the identification of highly penetrant, rare variations underpinning rare monogenic Mendelian disorders and birth defects (Bailey-Wilson and Wilson, 2011). Previously, several linkage workflows have helped in delineating/mapping the underpinning genes for a number of RDs in large multi-case families (Barrett, 2014). It is worth mentioning that molecular etiology of nearly 3,500 known RDs have been delineated primarily through conventional positional cloning methods based on linkage analysis and “homozygosity mapping” in which inheritance pattern of specific DNA markers such as single nucleotide variations (SNVs) and microsatellite repeats was used for ascertaining recombination events in extended multi-case pedigrees. Nevertheless, the remaining disorders have been refractory to these classical genetic screening methods for several reasons: locus heterogeneity, phenotypic heterogeneity, reduced penetrance, availability of only a small number of patients or families which may not be sufficient enough to attain a high-power study, and substantially reduced reproductive fitness in the patients due to early disease onset and severe effect (Lander and Botstein, 1987; Vink and Boomsma, 2002; Botstein and Risch, 2003; Ng et al., 2010c; Boycott et al., 2013). These classical approaches remain uninformative in case of spontaneous and non-inherited disorders and are expensive, labor intensive and time consuming.

### Next-Generation DNA Sequencing

Several recently emerged state-of-the-art molecular biological techniques such as chip-based DNA arrays and several “massively parallel” or high throughput NGS technologies has helped overcome the shortcomings of previously described studies. The initial information on the human genome came into limelight with completion of the Human Genome Project that mostly relied on the “hierarchical shotgun strategy” carried out through the classic Sanger biochemistry (Lander et al., 2001; Shendure et al., 2017). After 2–3 decades of gradual improvements in sequencing biochemistry and technology, NGS technologies have vastly outpaced our ability in a way that presently several samples can be sequenced simultaneously on a single platform (multiplexing) with a higher accuracy and lesser cost (Shendure et al., 2017). This has subsequently lead to an era of unprecedented productivity – that is, progressive accumulation and availability of genomic data of individuals (personal genomes or exomes) of genetically varied populations that has facilitated comprehensive understanding of several human diseases and their susceptibility among different population groups (Wang et al., 2013; Middha et al., 2015). Over the past decade, NGS has emerged as a leading player in the odyssey of finding the underpinning causes of several RDs. It has created a paradigm shift in clinical genetics through a relatively easier discovery of the underpinning genetic causes of a number of RDs at a much lesser cost, thereby, obtaining a precise delineation for many uncharacterized RDs cases (Sawyer et al., 2016; Nambot et al., 2018). Discovery of new disease-associated genes and novel genetic variations through different NGS platforms has efficiently reinvigorated our understanding on the etiology of several genetic disorders, especially those of rare Mendelian disorders, which has led to the development of disease-specific diagnostic procedures and therapeutics (Shendure et al., 2017).

Since the previous decade, NGS helped overcome challenges associated with traditional gene discovery approaches, made it easier to identify the underpinning genetic etiology of several RDs and provided insights into their distinct underpinning biological mechanisms (Shendure et al., 2017). Due to the wider span, intrinsic complexity and greater cost of whole genome sequencing (WGS), whole exome sequencing (WES) has relatively gained more popularity in the identification of RDs genetics since 2010. It is based on the sequence analysis of exome (the total protein-coding content of the genome) which represents about 1% of the human genome and is known to harbor nearly 85% of the genetic variations that have large effects on the human physiology and cause disease-related phenotype (Choi et al., 2009). With continuous refinements in the sequencing technology and having aided in discovery of more than 550 distinct novel disease-associated genes, WES has emerged as an amazing technological advancement that has enhanced our understanding of the structure and function of the human genome and the molecular pathways underpinning human development and disease biology (Boycott et al., 2013).

The initial proof-of concept for the potential role of WES in RDs diagnostics and research came into limelight with the identification of genes responsible for Kabuki Syndrome (OMIM: 147920) and Miller syndrome (OMIM: 263750). Using WES and targeted sequencing, the researchers at University of Washington had identified and reported distinct *MLL2* gene (currently known as *KMT2D* gene) and *DHODH* gene variants responsible for Kabuki Syndrome and Miller syndrome, respectively, in majority of the afflicted individuals (Ng et al., 2010a, b). Later, protein truncating *PRRT2* variations were identified in a familial case of Paroxysmal Kinsesigenic Dyskinesia through WES (Chen et al., 2011). Variations in *SMARCB1*, *SMARCA4*, *SMARCA2*, *SMARCE1*, *ARID1A*, and *ARID1B* genes encoding proteins belonging to SWI/SWF chromatin-remodeling complex were identified using WES and implicated in a rare congenital anomaly called Coffin-Siris syndrome (OMIM: 135900) (Tsurusaki et al., 2012). A case of ectrodactyly and lethal pulmonary acinar dysplasia associated with *FGFR2* variations in a 5 day old baby born to normal consanguineous was identified using WES (Barnett et al., 2016). Evidently, there has been a proportional flurry of reports at an accelerating rate on discovery of RDs-associated genes and novel variations which, in turn, has accelerated precise diagnosis of a number of suspected characterized as well as uncharacterized RDs since the advent of WES in 2010 (Yang et al., 2014; Shendure et al., 2017). WES has been successful in leading to precise diagnoses in an estimated 30–50% of RDs cases in clinical settings (Fresard and Montgomery, 2018). Despite, the molecular etiology of nearly one-third of RDs is still unknown and remains to be discovered (Boycott and Ardigo, 2018). WES is still not the gold-standard diagnostic approach for clinically uncharacterized diseases owing to its technical limitations that restrict the determination of variations in non-coding and/or regulatory genomic regions, structural variations and complex genetic mechanisms such as somatic mosaicism and gene imprinting underlying a substantial fraction of RDs (Fresard and Montgomery, 2018). Here, coming to rescue from the limitations of WES is another NGS technology, that is, the WGS. Besides the identification of SNVs, WGS is also capable of determining structural as well as epigenetic changes in the genome (Wang et al., 2015).

Success of NGS mostly relies on the accuracy of data mining tools used for the analysis of sequencing data. There is ample availability of raw NGS data processing and variant calling tools. Initially, processing of raw NGS data includes several steps such as quality check, adapter trimming, post-trimming quality check, PCR duplicate removal, alignment of sequencing reads to the reference genome and variant calling. FastQC is a tool used for checking quality reports of pre- and post-trimming sequencing data (Andrews, 2010). Trimmomatic is used for the initial quality trimming of the reads (Bolger et al., 2014). MarkDuplicates utility of Picard tool is used for PCR duplicates removal (Wysoker et al., 2013). Bowtie (Langmead et al., 2009) and Burrows-Wheeler Alignment (BWA) tool (Li and Durbin, 2009) are used for alignment of the reads to the reference genomes. Tools that are used for variant calling from NGS data include DISCOVAR (Weisenfeld et al., 2014), genome analysis toolkit (GATK) (Mckenna et al., 2010) for SNVs, DELLY (Rausch et al., 2012), GASV (Sindi et al., 2009), LUMPY (Layer et al., 2014) for Structural Variations, CoNIFER (Krumm et al., 2013), CONTRA (Li et al., 2012), XHMM (Fromer et al., 2012) for Copy Number Variations, Platypus (Rimmer et al., 2014) for SNVs, indels, repeat elements, *de novo* variations, and SAMtools (Li et al., 2009) for SNVs and short indels. These tools are capable of mining different types of variations from NGS data with a higher accuracy.

## Burden of Genetic Disorders in India

Population stratification in India has added to the country’s population diversity and gene pool. A high inbreeding rate in some specific Indian population clusters hint toward a relatively higher burden of specific genetic diseases and founder variations. India is, thus, considered as a unique hotspot of inherited genetic disorders and variations. With India’s recent accelerating clinical demographic switch to non-communicable diseases, congenital malformations/birth defects and genetic disorders have emerged as the major causes of mortality in the perinatal period (Verma and Bijarnia, 2002). A higher burden of inherited genetic disorders and variations highlights the importance of dissecting the genetic etiology and pathogenesis of several recessive disorders and complex diseases in India.

Brief accounts on the population stratification, inbreeding, genetic disorders, and genetic services in India are as follow:

### Population Architecture of India

India, the world’s second most populous country, holds the distinction of being the sixth largest home to more than one-sixth of the global human population (Aggarwal and Phadke, 2015). During prehistoric and historic times, the country has served as a major corridor for different migratory waves of anatomically modern humans (Majumder and Basu, 2014). These migratory events have significantly contributed to high heterogenic population stratification in the inhabiting Indian population groups in terms of their religious, socio-cultural, linguistic and racial backgrounds. In an evolutionary context, the population diversity in India has been considered as a result of admixture of multiple migratory populations and invaders belonging to the northwestern and eastern corners of the globe that had entered into the country by following land and coastal routes (Basu et al., 2016). Genetic studies have indicated that the modern Indian population is as an admixture of five large ancestral, genetically divergent, heterogeneous population groups. The ancestral groups comprising the Indian mainlanders include the “Ancestral North Indian (ANI),” “Ancestral South Indian (ASI),” “Ancestral Austro-Asiatic (AAA)” and “Ancestral Tibeto-Burman (ATB),” and a separate ancestral group named the “Ancient Ancestral South Indian (AASI) – related” for the people of the Andaman archipelago (Basu et al., 2016;Narasimhan et al., 2018). However, the current Indian population can also be categorized into four ethno-racial groups namely the “Australoids,” “Caucasoids,” “Mongoloids,” and “Negritos,” stratified into more than 4,000 anthropologically distinct population groups having their individual linguistic profiles (Aggarwal and Phadke, 2015). Based on religious-socio-cultural backgrounds, the Indian population is further sub-classified into different religious groups, castes and tribes. A vast majority (∼80%) of the Indian population comprises of the Hindu population groups which is further sub-divided into castes and sub-castes, about 8% is represented by the tribal populations while the rest of the population is comprised of other religious groups such as Muslims, Christians, Buddhists, Jews, Sikhs, and others (Indian Genome Variation Consortium, 2005).

### Inbreeding in India

The contemporary Indian population groups is an agglomeration of several thousands of separate endogamous groups (>50,000) residing in topographically alienated pockets, many of which have been in existence for at least 100 generations (Mcelreavey et al., 2005). These groups represent distinct conservative breeding pools in which marriages are usually restricted within same religion, caste and biraderi according to the customs dating back to some 3,000 years (Bittles et al., 2002). In India, the establishment of marital relationships among individual population groups is guided by different regulations which are usually based on their distinct religious-socio-cultural norms. For instance, the Hindu religious group is structured into several hierarchical socio-cultural groups called varnas (Brahmins, Kshatriyas, Vaishyas, Shudras) which are sub-divided into castes (or jatis) (Indian Genome Variation Consortium, 2005). The population groups based on varnas and castes are usually endogamous. Each caste group is sub-divided into patrilineal groups or sub-castes known as gotras, each representing an exogamous group. The tribal sections or the ancestor-worshippers are mainly endogamous (Indian Genome Variation Consortium, 2005).

Nevertheless, consanguinity is also practiced as a custom in some specific Indian population groups, with rate of consanguinity ranging between 20 and 30% (Bittles, 2002a). Among the Indian Hindus, non-uniform views pertaining to consanguinity subsist with more complex marriage regulations (Bittles and Black, 2010a). According to a general prohibition dating back to 200 BC, the majority Hindu population in the northern, eastern, and north-eastern states rigorously forbid consanguinity by avoiding same “gotra” marriage including those between kins and between a man and his father’s sister’s or mother’s sister’s or mother’s brother’s daughter; though a long tradition of first-cousin marital union, uncle-niece marriage and marriage between a man and his maternal uncle’s daughter is prevalent among Dravidian Hindus belonging to southern India and in most Christian denominations, mostly reported from rural communities and among the underprivileged (including the poorest, illiterate, and least educated) groups (Hussain and Bittles, 2000; Bittles, 2002b). First-cousin marriage, particularly between a man and his maternal uncle’s daughter, is generally preferred in Andhra Pradesh, Karnataka, and Tamil Nadu and in Kerala, Goa, and southern Maharashtra to a lesser extent (Bittles, 2002a). The Muslim religious group practice consanguinity at a higher rate with no comparable north-south distinction in consanguinity, as indicated in Table 1 (Bittles, 2002b). Although consanguineous marriages are forbidden in the Sikh religion, some minority Sikh groups in India appear to exercise flexibility in the observance of this proscription by allowing first- or second-cousin marriages (Table 1) (Bittles, 2002b).

**TABLE 1:** Rate of consanguineous marriages among various religious groups in India during 1992–1993 (Bittles, 2002b).

S. no.	Religion	Rate of consanguinity (%)	Mean coefficient of inbreeding (α)
1	Hindu	10.6	0.0068
2	Muslim	23.3	0.0141
3	Christian	10.3	0.0068
4	Sikh	1.5	0.0009
5	Jain	4.3	0.0024
6	Buddhist	17.1	0.0107
7	Others	8.7	0.0053

### India as a Trove of Genetic Disorders

Of the 350 million global estimate of RDs patients, India alone is a home to approximately 70 million (equating to 1 in 20) patients with any of the known progressive, life-threatening and chronically debilitating rare health anomalies which encompass a wide range of systemic disorders including immunodeficiency syndromes, blood disorders, skeletal disorders, neurological disorders, and many more (Kumar H. et al., 2017). This indicates that the cumulative burden of RDs is quite significant in India in comparison to the world average owing to its highly inbred population structure (Kumar H. et al., 2017). Unfortunately, there is no standard definition to describe the prevalence of a RD in India; though Organization for Rare Diseases India (ORDI) has suggested a threshold for defining a disease as rare if it afflicts 1 in 5,000 individuals in India (Rajasimha et al., 2014).

Religious restrictions compounded by the geographical isolation of some Indian habitats due to the country’s diverse topography have contributed significantly to a relatively higher rate of inbreeding (population-inbreeding coefficient of India = 0.00–0.20) and, thus, served as barriers to random mating and free gene flow leading to the distinct gene pool of the Indian sub-populations (Bittles et al., 2002). In parallel, there is also a relatively higher burden of specific diseases usually restricted and/or unique to specific Indian ethnic groups, sub-castes, tribes or clans under the influence of founder events that have occurred 50–100 generations back (Bittles, 2002a; Mcelreavey et al., 2005; Pradhan et al., 2011; Dixit et al., 2015). A list of some of the disease-associated founder variations determined from the Indian population has been indicated in Table 2. One of the biggest examples supporting the association of endogamy and community-specific disease burden in India is a highly endogamous Agarwal community. Genetic diseases such as Megalencephalic Leukodystrophy with sub-cortical cysts (OMIM#604004), Panthothenate Kinase-Associated Neurodegeneration with Brain Iron Accumulation (PKAN; OMIM#234200) and Spinocerebellar ataxia type 12 (OMIM#604326) associated with a number of founder variations in *CAPN3* (OMIM#114240), *MLC1* (OMIM#605908), *PANK2* (OMIM#606157), *PPP2R2B* (OMIM# 604325) genes, respectively, are frequently observed in this Indian community (Table 3). Genetic disorders reported from India are enlisted in Table 4. Besides a higher rate of inbreeding and founder effects in some Indian sub-population groups, other factors including lack of diagnostic, disease management and rehabilitation infrastructure in the country, its large population size and high birth rate have contributed significantly to a relatively higher incidence of recessive disorders in India (Verma, 2000; Verma and Bijarnia, 2002).

**TABLE 2:** A list of some of the disease-associated founder variations prevalent in India.

S. no.	Disorder	Gene	Variation	Region	References
1	Combined pituitary hormone deficiency	*PROP1*	c.112_124del	Indian subcontinent	Turton et al., 2005
2	Growth hormone deficiency	*GHRHR*	c.214G>T	Indian subcontinent	Wajnrajch et al., 2003
3	Haim-Munk syndrome and Papillon-Lefévre syndrome	*CTSC*	c.2127A>G	Immigrated Jews (from Cochin) in Israel	Hart et al., 2000
4	Hemophilia B	*F9*	c.316G>A	Gujarat	Quadros et al., 2009
5	Oculocutaneous albinism type 1	*TYR*	c.832C>T	Tili community, West Bengal	Chaki et al., 2005
6	Progressive pseudorheumatoid dysplasia	*WISP3*	c.1010G>A	South India	Dalal et al., 2012
7	Tay-Sachs disease	*HEXA*	c.1385A>T	Gujarat	Mistri et al., 2012
8	Tricho-hepato-enteric syndrome	*TTC37*	c.2808G>A	Gujarat	Kotecha et al., 2012
9	Von Willebrand disease	*VWF*	c.6187C>T	Kachi Modh Ghanchi community in Gujarat	Kasatkar et al., 2013
10	Werner syndrome	*WRN*	c.561A>G	Kerala	Saha et al., 2013

**TABLE 3:** A table representing some of the founder variations prevalent in a highly endogamous Indian Agrawal community.

S. no.	Disorder	Gene	Variation	References
1	LGMD2A	*CAPN3*	c.2338G>C	Ankala et al., 2013, 2015
			c.2099-1G>T	
2	Megalencephalic leukodystrophy with sub-cortical cysts	*MLC1*	c.135dupC	Gorospe et al., 2004
3	PKAN	*PANK2*	c.125_216insA	Udani et al., 2017
4	Spinocerebellar ataxia type 12	*PPP2R2B*	CAG expansion	Sinha, 2005

**TABLE 4:** The estimated rate of incidence of common genetic disorders in India (Ankala et al., 2015).

S. no.	Genetic disorder	Estimated rate of incidence
1	β-thalassemia	1:1,500
2	Congenital hypothyroidism	1:1,700
3	G6PD deficiency (South India)	1:2,200
4	Congenital adrenal hyperplasia	1:2, 2,600
5	α-thalassemia	1:3,500
6	Amino acid disorders	1:3,600
7	Galactosemia	1:10,300
8	Phenylketonuria	1:18,300

### Status of RDs-Genetic Services in India

Like other developing countries, India too faces a number of challenges in dealing with RDs as a major public health issue. There is a lack of true, reliable quantitative data on individual as well as cumulative prevalence of RDs at the national and regional level and the epidemiology of associated morbidity and mortality. These factors have further impeded the RDs-related reliable cost estimations and the implementation of relevant research and development programs for RDs therapeutic management in the country. Other challenges include lack of availability of specialized medical personnel, molecular diagnostic infrastructure, and standard therapeutic drugs and protocols committed to understanding and management of RDs. The genetic background of the Indian population is also not well understood due to its under-representation in the major global genomic studies.

Several individual research groups, pharmaceutical companies and patient assistance organizations and their charitable programs are consistently working on advocacy of the importance of RDs diagnosis, research and drug development and framing of a national RDs policy in India. These include ORDI, Indian Organization for Rare Diseases (I-ORD), Foundation for Research on Rare Diseases and Disorders- Rare Diseases India (FRRDD-RDI), Open Platform for Rare Diseases (OPFORD), Genomics for Understanding Rare Diseases India Alliance Network (GUaRDIAN) and many more (Rajasimha et al., 2014). Several case reports for distinct RDs from India have been published during the recent years. These include a series of reports on accurate diagnosis of RDs cases including Acid Sphingomyelinase (ASM)-Deficient Niemann-Pick Disease, Allgrove or Triple A (AAA) syndrome, Ethylmalonic Encephalopathy, Fanconi-Bickel Syndrome, Fructose-1,6-biphophatase Deficiency, Homozygous familial hypercholesterolemia, Mucopolysaccharidoses type I and type II, Rhizomelic chondroplasia punctata type 1, progressive pseudorheumatoid dysplasia and many more using Sanger Sequencing of disease-associated genes (Phadke et al., 2010; Dalal et al., 2012; Kehar et al., 2014; Bijarnia-Mahay et al., 2016; Ranganath et al., 2016; Setia et al., 2016; Uttarilli et al., 2016; Angural et al., 2018; Bhai et al., 2018). Besides, there are several case reports indicating the major application of NGS technology in RDs diagnostics including that of ichthyosis, rare syndromes of mineralocorticoid excess, dystrophic epidermolysis bullosa, cone dystrophy, sporadic acrokeratosis verruciformis, Dowling-Degos disease, Spastic Paraplegia 79 and many more, (Gupta et al., 2015, 2016, 2017; Karuthedath Vellarikkal et al., 2016; Narayanan et al., 2016; Das Bhowmik et al., 2018; Virmani et al., 2018). Many of these diagnosed RDs are so rare that they have been reported either distinctly or as spectrum disorders for the first time from India. In this context, there is a dire need of conducting genetic screening of the uncharacterized RDs patients and their families and population genetic studies for elucidating the load of pathogenic variations harbored by the Indian population and establishing its own population-based genetic variation database.

## Jammu and Kashmir: a Model Population for Studying Rare Genetic Disorders

Despite continuous global efforts, a very little attention has been paid to the most challenging health issue of RDs in India with special reference to region like J&K. J&K is located in the northern part of the Indian sub-continent in the vicinity of the Karakoram and westernmost Himalayan mountain ranges (outer hills, middle Himalayas, and inner Himalayas) (Bhasin and Nag, 2002b). The region is being bordered by countries such as Pakistan in the west, Afghanistan in the north-west and China in the north-east (Bhasin and Nag, 2002a). Topographically, J&K is divided into three main isolated divisions, namely Jammu, Kashmir, and Ladakh (Sharma et al., 2018). The region is characterized by tough mountainous terrains that have rendered geographical isolation of its heterogeneous population. J&K has been consistently under geo-political turbulences and terrorism and, in general, has low resources including basic facilities of education, feeding, healthcare, electricity, sanitation, and transportation. Owing to geographical isolation and religious socio-cultural norms, majority of the inhabiting population is highly endogamous and consanguineous.

A huge burden of RDs has been suspected in J&K. According to the information available from Rare Diseases India Organization, more than 0.7 million individuals from J&K are likely suffering from RDs. Nevertheless, this information appears to be vague due to lack of a centralized patient-registry which has resulted in an unfortunate lack of accurate epidemiological data on distinct RDs prevalent in the region. Adding further to this is the lack of appropriate clinical information on these diseases, lack of awareness among the general population and basic facilities such as tertiary care hospitals, medical personnel, diagnostic facilities, and scanty R&D centers. Although there is a magnanimous quantitative burden of RDs in J&K, but these have mostly remained clinically ignored owing to aforementioned issues. Unfortunately, the population groups of J&K have also largely remained under-represented in the previous Indian genetic studies until recently, resulting in a lack of information on the genetic make-up of the J&K population strata and their disease heritage. In context with the region’s geographical topography and higher inbreeding rate, the unexplored cases of genetic disorders are likely to be associated with founder events unique to different population groups or families which may also hold clues for their evolutionary perspectives. The genetic assessment of the affected individuals from the region is, thus, a need-of-the-hour and essential for the development of appropriate therapeutic interventions. This would aid in genetic counseling and management of the reported genetic diseases.

In this section of the review, brief accounts on the population architecture of J&K, burden of genetic disorders and genetic studies conducted in the region have been provided.

### Population Architecture of J&K

The population of J&K is typically heterogeneous with many anthropologically well-defined distinct ethnic and religious groups. The ethnic groups residing in J&K mainly include Arghuns, Bakerwals, Baltis, Bedas, Bodhs, Brokpas, Changpas, Dogras, Garras, Gujjars, Harijans, Kashmiris (Pandits and Muslims), Khatris, Kishtwaris, Ladakhis, Mahajans, Mons, Paharis and Purigpas (Bhasin and Nag, 2002a). The major religious groups of J&K include the Buddhists, Christians, Hindus, Jains, Muslims, Sikhs and others. According to the Population Census of India – 2011, Muslims (68.31%) constitute the major inhabiting population strata in J&K, followed by Hindus (28.44%), Sikhs (1.87%), Christians (0.28%), Jains (0.02%) and others (0.17%). The contemporary population of J&K speaks languages belonging to three different linguistic families – the Indo-European, Tibeto-Burman (in Ladakh) and various dialect of Dardic (in Kashmir) from Indo-Aryan language group (Sharma et al., 2018). These population groups usually reside in small, geographically and socially isolated pockets since many centuries and are variably reinforced by their societal or religious customs to practice endogamy or consanguinity. Endogamy is preferred by almost all the population groups of J&K, whereas the consanguinity is mostly preferred by the Muslim population groups (Bhasin and Nag, 2002b). A study from the Rajouri and Poonch areas of J&K has indicated that the rate of consanguinity is quite high among the native Muslim populations which account nearly 35–50% with nearly 70–80% of the inbreeding occurring among first cousins (Fareed and Afzal, 2014a, 2017). However, the reported figures were limited to the Muslim population of only two areas in J&K and might vary between different regions and communities.

### Burden of Genetic Diseases in J&K

The population stratification of J&K holds a high significance in the historical, religious, socio-cultural and linguistic diversification of the Indian population. It has been suggested that various pre-historic and historic events of migrations and immigrations toward the Indian sub-continent have occurred through J&K along the north-eastern and north-western routes (Bhasin and Nag, 2002a; Pandith et al., 2015; Sharma et al., 2018). Intense endogamy within the population groups of J&K has resulted in restricted gene flow and genetic isolation for several centuries, thus, making them unique in terms of their gene pool and disease heritage. It is known that genetically isolated and highly endogamous population groups have higher levels of genetic homozygosity and, thus, are relatively more prone to have a higher burden of genetic disorders, especially recessive RDs (Woods et al., 2006; Bittles and Black, 2010). In context to J&K, this fact could be supported by a study conducted on 995 individuals belonging to six Muslim population groups (including Gujjars and Bakerwals, Khans, Maliks, Mirs, Mughals, and Syed) from Rajouri and Pooch areas in J&K which has indicated a relatively higher level of homozygosity for Rhesus factor alleles (Fareed et al., 2014).

Since past few years, a number of suspected cases of genetic disorders (including new/known monogenic diseases and other known genetic diseases with atypical clinical features) have been reported from J&K region. Cases of chromosomal genetic disorders (such as Down Syndrome, Turner Syndrome, Klinefelter Syndrome, Patau Syndrome; Table 5), anemias, blood disorders (including Thalassemia), congenital anomalies, disorders of sex development, metabolic disorders like G6PD deficiency, neurological disorders (Table 6) and others are frequently reported in J&K (Razdan et al., 1994; Kumar et al., 2010; Upma et al., 2010; Vasudev and Sawhney, 2014; Ara et al., 2018; Dar et al., 2018; Hockham et al., 2018). The detrimental effects of consanguinity and inbreeding depression on child health and mortality, cognitive behavior and fertility and an increased risk of cardiovascular diseases in small population groups from J&K has also been reported (Bhasin and Nag, 2002b; Fareed and Afzal, 2014a, b, 2016; Fareed et al., 2017). However, the J&K population has been largely under-represented in the surveys/screening studies conducted in context with disease incidence. These studies were usually restricted to small regional pockets in different areas of J&K and, therefore, have created a huge gap in the literature on incidence of the prevalent diseases.

**TABLE 5:** Figures on the reported cases of chromosomal anomalies in J&K (Kumar et al., 2010).

S. no.	Chromosomal anomaly	Number of effected individuals	Percentage/Frequency
1	Down syndrome	80	49.7/0.497
2	Delayed milestones	50	31.1/0.311
3	Turner syndrome	18	11.2/0.112
4	Klinefelter syndrome	11	6.8/0.068
5	Patau syndrome	2	1.2/0.012
	Total	161	100/1

**TABLE 6:** Prevalence of various neurological diseases, as reported in rural Kashmir during 1986 (Razdan et al., 1994).

S. no.	Neurological diseases	Prevalence
1	Stroke	1.43/1,000
2	Seizures	2.47/1,000
3	Mental retardation	2.09/1,000
4	Poliomyelitis	2.18/1,000
5	Cerebral palsy	1.24/1,000
6	Parkinsonism	1.41/10,000
7	Deaf mutism	1.63/1,000
8	Peripheral neural diseases	2.99/10,000
9	Other extra-pyramidal movements	1.73/10,000
10	Spinal cord lesions	2.36/10,000
11	Tuberous sclerosis	0.62/10,000
12	Muscle diseases	0.79/10,000
13	Neurofibromatosis	0.31/10,000

There are some curious cases of certain clinical conditions that are highly prevalent in different hotspots or villages located in isolated remote areas of J&K. These reports indicate a higher burden of some diseases in the region restricted to individual villages or families. Information on these cases has been mostly portrayed over narrow approaches in the form of generalized or journalistic information. Summarized reports on some of these have been provided as follows:

**Report 1 – The Village of Silence, J&K:** Dadhkai village of Bhalessa, often known as the “Village of Silence,” in Doda area has been reported to have a high incidence of hearing loss. As per the reports (both journalistic as well as literary), this hamlet is inhabited by more than 2,500 individuals with over 95 members being deaf and mute. At least there is one member in each family who can neither hear or is mute. However, it has been reported that there is an increase in number of affected individuals in the region during the past decades. The village is located in a remote isolated area characterized by a very tough terrain in the Himalayan Mountains. It lacks basic facilities of nutrition, healthcare, immunization and rehabilitation, education, electricity, roads, and transportation. It is connected to the mainland through a foot-bridge. Some of the individuals have been found to be iodine- and salt-deficient by the clinicians. The residents of the village are of the opinion that the community might have been under some curse. The majority of the population in Dadhkai village belongs to a scheduled tribe “Gujjar” community of Muslim religion. However, given impetus to prolonged endogamous practices among this community, the clinical condition must have genetic origin and, thus, attained a relatively high incidence in the village. Pedigrees of four families from Dadhkai are already available in the literature (Raina et al., 2017).
**Report 2 – Arai village in Poonch, J&K:** There are reports on a mysterious skeletal disease highly prevalent in Arai, a cluster of three villages, in Mandi area of Poonch – J&K. The symptoms of the disease usually appear between 4 and 8 years of age and progress in severity with advancing age. The affected individuals develop enlarged joints that look knobby in appearance, pain in joints, gait disturbance, abnormal posture, and short stature. More than 100 individuals over two extended families in the village have been rendered crippled for their life with this disease. Arai is located in the remote mountainous terrains of the Himalayas and has remained isolated due to lack of transport facilities until the roads were laid recently in the village area. The region also lacks basic facilities of education, electricity, healthcare, and sanitation. The residents of Arai are economically poor. Owing to their incurable ailment and lack of medical awareness, local belief of a curse resulting in the skeletal disorder in families has remained for decades and, thus, families have given up on medical consultations. Recent extensive efforts and a study reported from Arai village characterized the disorder as progressive pseudorheumatoid dysplasia (PPD), an autosomal recessive genetic disease with variants in gene *WISP3* as the cause (Rai et al., 2016).
**Report 3 – Village of deaf and mute in Paralkot, J&K:** In a remote village named Paralkot, about 80% of the population has been reported to be deaf and mute. The village falls in Sawjian area of Mandi sub-division, Poonch, J&K near the Line of Control (LoC), and lacks basic facilities of education, health-care and transportation. Majority of the inhabiting population is poor and are laborers. The villagers have different telltales regarding their clinical condition. Some believe that the person who sees the fairies residing in a nearby mountain become deaf and mute, while others believe there are evil spirits in the area or some curse shadowed over their families. It has been reported that owing to their clinical condition, about 30 families from this village had migrated to Pakistan occupied Kashmir (PoK) during 1990–1991. However, the villagers marry their close relatives within the same village. Owing to their endogamous background, there could be a likely genetic cause for their hearing disability as indicated by our preliminary (unpublished) findings.

There are further series of case reports on RDs including Ellis-van Creveld Syndrome, Epidermolysis Bullosa, Fabry’s disease, Fahr’s disease, Hereditary Stomatocytosis, Holt-Oram Syndrome, Ollier’s disease, different types of Porphyria, Rogers Syndrome, Wolfram syndrome, congenital anomalies, and many more from different regions of J&K (Bhat J. I. et al., 2010; Bhat Y. J. et al., 2010; Bhat et al., 2015; Qayoom et al., 2010; Ganie et al., 2011, 2012; Majid et al., 2012; Shoib et al., 2012; Hassan and Keen, 2013; Rasool et al., 2015; Wani et al., 2016; Kumar S. et al., 2017; Nazir and Chalkoo, 2017; Ilyas et al., 2019). Most of these are merely presented as case reports and the underpinning molecular etiology of many of the reported as well as unreported RDs cases from J&K have been due for years. Further there is a likelihood of more under-represented or yet to be identified RDs cases from J&K.

### RDs-Associated Genetic Studies Conducted in the Region so Far

With advances in the genome research technologies, researchers have been recently successful in delineating some cases of RDs prevalent in J&K. The findings of these studies have immense contribution in expanding the genotype-phenotype and geographical spectrum of the reported RDs. Interestingly, these reports have been variably reported from different regions across the globe in high numbers from consanguineous/endogamous population groups in association with specific founder events. So far, these reported RDs cases have been reported for the first time from J&K in association with variations restricted to individual families or population groups of which a few indicates the presence of likely founder events.

Two studies based on *CFTR* (OMIM# 602421) variation analysis by Sher-e-Kashmir Institute of Medical Sciences, Kashmir in the Kashmiri population suspected with Cystic Fibrosis (OMIM# 219700) has revealed disease’s association with highly frequent *CFTR*. ΔF508 (c.1521_1523delCTT; p.Phe508del) and *CFTR*. 3,849+10 kb C > T variations (Kawoosa et al., 2014; Pandith et al., 2015). The major limitation of these studies was that these were based on screening of only two *CFTR* variations in limited sample size. Considering the heterogeneity of the J&K population, it would have been highly informative if these studies could have screened the whole *CFTR* gene. Nevertheless, it is pertinent to mention that until these molecular studies had been conducted, Cystic Fibrosis was earlier considered to be uncommon in J&K. A team of researchers from Jammu had conducted a pilot study on screening of coding region exon 2 of *GJB2* gene (OMIM# 121011) in randomly selected 17 affected individuals from the previously mentioned Dadhkai village (Village of Silence) in Doda – J&K (Razdan et al., 2012). Through this study, *GJB2* variations in only 4 out 17 subjects were detected which included p.G12V, p.L6L, p.R165W, p.L214P, and Del T at nt 636 variations. The study could have benefitted and informative if screening of other genes associated with hearing loss been performed. A further interesting study on eight families (one large and seven small families) representing about 50% of the affected individuals from the same village was conducted jointly by the Department of Biotechnology in University of Kashmir (Kashmir), Molecular Biology and Genetics Unit in Jawaharlal Nehru Centre for Advanced Scientific Research (Bengaluru) and Department of Audiology in Ali Yavar Jung National Institute for the Hearing Handicapped (Mumbai) (Pandey et al., 2017). The families were screened using methods like Genome-wide scan and linkage analysis, mutation analysis of *OTOF* (OMIM# 603681), *Cx26*or*GJB2*, *TMIE* (OMIM# 607237), *CLDN14* (OMIM# 605608), *SLC26A4* (OMIM# 605646), *TMPRSS3* (OMIM# 605511), *TMC1* (OMIM# 606706), and *USH1C* (OMIM# 605242) genes. The findings of this study suggested genetic heterogeneity underpinning hearing loss among inhabitants of the highly endogamous Dadhkai village. Genome-wide scan and linkage analysis of the large family extending upto six generations mapped deafness to a chromosome loci 2p24-p22. The findings of the mutation analysis indicated a novel, founder variation NM_194248.2:c.2122C>T (NP_919224.1:p.R708^∗^) in exon 18 of *OTOF* gene, NM_144492.2:c.254T>A (NP_652763.1:p.V85D) in exon 7 of *CLDN14* gene (a founder variation with origin in Pakistan) and a novel NM_00441.1:c.1668T>A (NP_000432.1:p.Y556^∗^) in exon 16 of *SLC26A4* gene causing hearing loss in 94% of the cases genetically screened through this study. Furthermore, an unidentified underpinning genetic cause in one of the families was suspected to be the fourth cause.

A case of Autosomal Recessive Spastic Ataxia of Charlevoix-Saguenay (ARSACS; OMIM# 270550) in individual born to consanguineous parents of tribal origin from Rajouri area of J&K has been recently delineated through WES and reported in a reputed peer-reviewed journal (Kuchay et al., 2019). The study identified a novel, frame-shift variation NM_014363: c.8605delT (p.Cys2869ValfsTer15) in exon 10 of *SACS* gene (OMIM# 604490), a well known causative gene of ARSACS. The proband was found to be homozygous for the reported variation, whereas his consanguineous parents were carriers.

Our research group (Human Genetics Research Group) at Shri Mata Vaishno Devi University, Katra, J&K – India is actively engaged in elucidating the underlying genetic causes for various complex disorders including type 2 Diabetes, Scoliosis as well as RDs and understanding the evolutionary perspectives of the population of J&K. In context with RDs, we have collected clinical information and samples from 60 distinct extended families suspected with uncharacterized genetic disorders from different regions of J&K. Of these recruited families, the exact genetic etiology of three distinct RDs have been determined and reported recently. The Arai village with a high incidence of a mysterious skeletal disorder (as mentioned earlier) was identified as a rare skeletal disorder “Progressive Pseudorheumatoid Dysplasia” (PPD; OMIM# 208230) using WES by our research group (Rai et al., 2016). Both affected as well as unaffected members of two highly extended consanguineous families in Arai village were recruited for their genetic screening. Through WES in three members (affected siblings and their distant uncle) belonging to one of the families revealed two co-segregating, highly autosomal recessive variations NM_003880.3:c.156C>A (NP_003871.1:p.Cys52^∗^; rs121908901) and NM_003880.3:c.248G>A (NP_003871.1:p.Gly83Glu; rs147337485) in exon 3 of *WISP3* gene (OMIM# 603400). These variations are already known to co-segregate with the disease phenotype in some families belonging to different parts of the world and might be an outcome in a region as a founder event, most likely of a middle-eastern origin (Delague et al., 2005). Both of these variations have also been reported in some South Indian families (Dalal et al., 2012). However, these variations were not found in the other recruited family for which sequencing of whole *WISP3* gene was carried out and it was found that the second family is harboring a novel, autosomal recessive, splice-site variation NM_003880.3:c.643+1G>A (rs879255273) at the *WISP3* exon 4 – intron 4 junction. Interestingly, PPD is a rare disorder having a prevalence of 1 in 1 million individuals in the United Kingdom, but have attained a higher prevalence in Arai village (upto 1000 times) due to the residing community’s consanguineous marital practices (Wynne-Davies et al., 1982). This was the first ever study from the region that exploited a NGS technique (WES) for the identification and characterization of an unknown disease.

In an independent study using a PCR-based Direct Sanger Sequencing Strategy, our research group has identified an autosomal recessive variant NM_153638.3:c.1069C>T (NP_705902.2:p.Arg357Trp; rs753376100) located in exon 3 of *PANK2* gene in a clinically suspected familial case of neurodegenerative disorder “Pantothenate Kinase-Associated Neurodegeneration” (PKAN; OMIM# 234000) (Angural et al., 2017). PKAN is a progressive neurodegenerative disorder characterized by an abnormal accumulation of iron in the basal ganglia in brain and extra-pyramidal manifestation (Hayflick et al., 2003). Thus, through this study the suspected PKAN diagnosis among two affected siblings, belonging to a remote village in Doda – J&K, was confirmed nearly within a decade of their preliminary diagnosis. The variation identified is located at a highly conserved region in codon 357 of hPank2 protein and predicted to be pathogenic through *in silico* pathogenicity prediction tools. Through a comparative molecular dynamics study of the wild-type and variant hPank2 protein models, it was observed that the reported variation has rendered rigidity to the other highly dynamic protein structure which might have caused a functional compromise in hPank2 molecules (Angural et al., 2017).

Recently, our group has also successfully identified an uncharacterized neurological case in a 9 years old boy as an atypical form of Leigh Syndrome (LS; OMIM# 256000) through Whole Mitochondrial Genome Sequencing, and was found to be associated with a novel heteroplasmic *MTP6* gene (OMIM# 516060) variant m.8936T>A (Angural et al., 2019). LS is a progressive neurodegenerative disorder of infancy or early childhood with a clinical and genetically heterogeneous background (Sofou et al., 2014). The proband in this study depicted an atypical feature of calcification in basal ganglia which was reported for the first time through this study.

## Bottom-Up Approach

**FIGURE 1:**
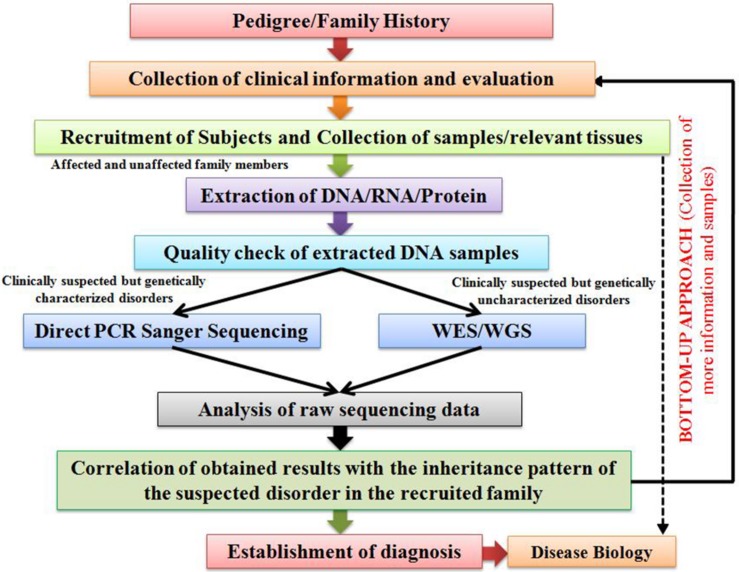
An illustration outlining the proposed “Bottom-up Approach” for the characterization of genetic disorders.

For carrying out genetic screening of cases of suspected genetic disorders, our research group has adopted the “Bottom-up Approach.” This approach is highly efficient in delineating the genetic etiology of various genetic disorders, especially RDs, prevalent in different global population groups. An outline of the designed Bottom-up Approach has been provided in Figure 1. The strategy begins with the collection of clinical information of the patient and his/her family’s clinical history in the form of a pedigree. This is followed by a strategic collection of samples (blood, saliva, and tissue) from the patients and some of their unaffected family members, so that a comparative genetic screening is conducted. Based on the clinical suspicion and prior knowledge on the molecular etiology of the suspected disorders, two distinct approaches are considered for the genetic screening. In case of clinically suspected but genetically characterized RDs, targeted screening of known disease-associated candidate genes through a PCR-based Direct Sanger Sequencing approach (candidate gene screening) is suggested. On the other hand, WES approach is adopted in case of clinically suspected but genetically uncharacterized RDs, the results of which are further validated through a targeted PCR-based Direct Sanger Sequencing approach to screen the identified variations in the recruited subjects. After rigorous analyses and interpretation of the raw sequencing data through various tools, the identified variations should be further analyzed for their plausible pathogenicity through various online *in silico* prediction tools, the brief details of which have been provided in Table 1. The plausible pathogenic variations are then marked for establishing their correlation with the clinical phenotype of the patients followed by analyses of their co-segregation with the suspected disorder in the recruited family. However, the final proof for variant-disease phenotype correlation is obtained through molecular functional studies based on DNA-RNA, DNA-Protein, RNA–RNA, and protein–protein interactions and expression studies dealing with the elucidation of query variation in a gene in model organisms. Once a variation is identified as pathogenic, a “Bottom-up Approach” is then followed which includes collection of more clinical information of the patient and his/her affected family members, and a comprehensive clinical evaluation in order to establish a differential diagnosis of the disease, followed by recruitment and genetic screening of other family individuals (including both affected as well as unaffected) for assessing the carrier frequency of the identified genetic variation.

## Conclusion

RDs are an important public health issue which needs to be overcome. To address RDs-associated challenges, a drive toward universal health coverage to fulfill RDs patients’ needs is required along with investment of public and government (national or international) funding in fundamental biomedical research for understanding the disease etiology, discovery of novel diagnostic biomarkers and therapeutic targets, and development of personalized intervention strategies for individual RDs patients. In promoting RDs-related R&D, significant progress has been made across the globe in recent years and many opportunities have been developed to build on the successful programs, projects and collaborations. For several RDs, remarkable fundamental research into the disease process has increased our understanding of RDs patho-physiology and led to development of suitable orphan drugs, healthcare innovation, and therapeutic interventions. Despite, there are still several hurdles in RDs research and healthcare and more emphasis is required to support appropriate RDs-related R&D and policy programs within individual countries so that all global patients would have equal access to therapeutic interventions. It is an irony that unlike other developing countries, India is lagging behind in context to regulation of RDs-based R&D due to several key issues that needs to be urgently addressed. The precise delineation of distinct RDs is possible by a meticulous clinical evaluation of the patients and their genetic screening. Recognition of carriers harboring clinically pathogenic genetic variations is important as to provide proper genetic counseling to the suspected individuals/families and an appropriate management of the disorder in an affected individual in a timely manner. For this purpose, we propose a highly potential workflow named “Bottom-up Approach” which could possibly aid in addressing this challenge not only for the Indian population, but for several other endogamous/consanguineous population groups existing across the globe. We further propose that the heterogeneous population of J&K could inspire future genetic studies and could serve as an interesting population-model for the same purpose. Besides, strong legislative policies and initiatives are also required from government and other institutions for carrying out RDs-related research.

## Future Perspectives

Although the main source of information on RDs support and research groups for the patients and their families remains the internet, yet national RDs support websites are still needed in many countries (Cullen, 2002; Lasker et al., 2005; Zurynski et al., 2008). To address the clinical challenges associated with RDs, it becomes imperative that different sources of clinical information and the clinical infrastructure should be updated regularly. A separate course on Clinical Genetics must be included in the academic curriculum of medical students in order to provide them knowledge on the basic concepts of Genetics and its applications in human health.

In context to the limitations of WES, it becomes imperative to use WGS and other “omics” platform as an alternative for determining the underpinning complex molecular etiology of RDs. This should also be accompanied with the development of innovative approaches that could possibly maintain and accelerate the current pace of clinical as well as genetic discoveries and inform future therapeutic developments.

We also emphasize that genetic screening of suspected population groups in J&K through the “Bottom-up Approach” based on the state-of-the-art biological techniques in amalgamation with clinical expertise should be carried out. In order to ascertain the genetic profile of the J&K population and the burden of carriers harboring pathogenic variations, a baseline database targeting each and every endogamous group from the region needs to be created, a daunting task undertaken by our Human Genetics Research Group at SMVDU but coming out with promising outcomes.

## Author Contributions

AA and ASp primarily wrote the manuscript and prepared the figure and tables. SS and ER critically edited the manuscript, planned various studies, and developed procedures and execution pipelines. AA, ASp, AM, VV, ASh, PK, MD, KP, ER, and SS were involved in carrying out various studies. MD facilitated Sanger Sequencing at DNA Sequencing Facility, University of Jammu.

## Conflict of Interest

SS has recently founded as a Chief Scientific Advisor a start-up “Biodroid Innovations Pvt. Ltd.,” involved in developing genetic and Med tech solutions. The remaining authors declare that the research was conducted in the absence of any commercial or financial relationships that could be construed as a potential conflict of interest.

## Footnotes

1 https://www.omim.org/

2 https://www.orpha.net/consor/cgi-bin/index.php
